# Adaptive Integrated Navigation Algorithm Based on Interactive Filter

**DOI:** 10.3390/s25154562

**Published:** 2025-07-23

**Authors:** Bin Zhao, Chunlei Gao, Hui Xia, Jinxia Han, Ying Zhu

**Affiliations:** 1 School of Marine and Electrical Engineering, Jiangsu Maritime Institute, Nanjing 211100, China; mirrorxh@163.com (H.X.); hanjinxiadream@163.com (J.H.); 2 Jincheng College, Nanjing University of Aeronautics and Astronautics, Nanjing 211100, China; gcl-12@163.com; 3 School of Electrical and Power Engineering, Hohai University, Nanjing 211100, China; yingzhu@hhu.edu.cn

**Keywords:** estimation accuracy, integrated navigation, interactive robust filter, smooth variable structure filter, strong tracking filter

## Abstract

To address the diverse requirements of accuracy and robustness in integrated navigation for unmanned aerial vehicles, an interactive robust filter algorithm that integrates the interactive multiple model concept and leverages the complementary applicability of the strong tracking filter and the smooth variable structure filter is proposed. The algorithm operates as follows: the strong tracking filter, along with the smooth variable structure filter, operates side by side with distinct models. During the filter process, the likelihood function is utilized to update the filter probabilities and determine the weights for each one of the filters. Input interaction, coupled with output fusion, is then carried out. The results of the experiments validate that the presented interactive filter algorithm significantly reduces estimation errors. When confronted with complex, dynamic noise environments and system uncertainties, it retains high-precision state estimation while demonstrating markedly improved robustness. The proposed interactive robust filter algorithm is compared against the strong tracking filter, smooth variable structure filter, and strong tracking smooth filter. Taking the strong tracking smooth filter, which has the highest accuracy among the three, as the reference baseline, the presented interactive robust filter algorithm achieves over 16% improvement in velocity accuracy and over 40% improvement in position accuracy.

## 1. Introduction

Unmanned aerial vehicles (UAVs), as autonomous navigation aircraft without crew, have many advantages such as flexible operation, no fear of casualties, and good concealment [1]. Fixed-wing UAVs are widely used in the military field due to their advantages of high flight speed, long endurance time, and strong load-carrying capacity. They have important strategic significance and are among key research focuses in the current UAV field [2]. Autonomous navigation, as the operational core of UAVs, ensures their safe and stable flight and their ability to fulfill mission tasks [3]. It is also a core and key technology in UAV development.

To meet the requirements of high precision, high stability, and high autonomy for fixed-wing UAV navigation, UAVs are mainly equipped with multiple navigation systems such as the strapdown inertial navigation system (SINS) and the Global Navigation Satellite System (GNSS). SINS is characterized by high autonomy and strong anti-interference capability, which makes it the core of the UAV navigation system. Navigation-grade strapdown inertial navigation systems have been applied to large fixed-wing UAVs successfully [4]. However, SINS errors accumulate gradually over time, with the altitude channel being particularly unstable. In contrast, the GNSS offers high long-term accuracy; however, it is susceptible to environmental and man-made interference, which results in degraded positioning and velocity measurement accuracy. A single navigation system is insufficient to meet the navigation requirements of UAVs. Therefore, it is necessary to fully utilize the measurements from the airborne navigation systems to achieve complementary advantages and conduct in-depth research on robust filter algorithms with wide applicability for information fusion [5]. Based on the complementary characteristics between SINS and GNSS, the SINS/GNSS integrated navigation has taken the dominant position in airborne integrated navigation [6].

To address the issue of the reduced accuracy of the conventional Kalman filter, caused by system modeling and noise uncertainties, the Strong Tracking Filer (STF) algorithm has been proposed and applied to data fusion [7,8]. Moreover, the STF strategy has been further integrated with various application contexts and filter methods. A strong tracking filter module is introduced to the track-by-detection framework for solving the sudden change problem of the target, and experimental results show that the presented approach demonstrates robust stability in noisy environments [9]. The strong tracking filter theory is incorporated into the nonlinear filter to develop a multiple-fading-factor square root cubature Kalman filter approach for neural network parameter training, which reduces training time and enhances convergence rate [10]. An adaptive filter integrating the Sage–Husa and strong tracking filter algorithms, which effectively enhances navigation accuracy, is studied [11]. The STF and its extended algorithms are proficient in tracking measurement information and exhibit good robustness against uncertain system noises. However, during the aerial missions of UAVs, the GNSS signals are vulnerable to environmental interference, resulting in uncertainties in the measurement noise. When the measurement information is inaccurate, the STF will still track rapidly, leading to more significant state estimation errors.

The Smooth Variable Structure Filter (SVSF), a model-based robust filter method developed by the Habibi team [12], mitigates performance degradation in the conventional Kalman filter induced by model uncertainties, interferences, and other factors. This method ensures that the state estimation converges to the interval of true value by means of the discontinuous gain in the form of a variable structure, and it exhibits good robust characteristics against bounded model and noise uncertainties [13]. Over the past few years, its application in the field of inertial integrated navigation has gradually expanded. An intensive exploration of SVSF theory is carried out and is applied to the SINS initial alignment [14]. The GPS receiver-derived attitude is integrated with three-axis gyroscope measurements via a robust SVSF, and simulation results validate the robustness of the proposed method [15]. However, this algorithm is essentially a suboptimal filter. While enhancing robustness, it sacrifices part of the estimation accuracy.

A combined smoothing variable structure–Kalman filter algorithm is proposed [16,17]: where the smoothing bounded layer width is less than preset threshold, the Kalman filter algorithm is adopted; otherwise, the smoothing variable structure filter is used. This method combines the Kalman filter’s accuracy and the smoothing variable structure filter’s robustness. However, the threshold of the bounded layer is set manually, and it often fails to effectively comply with real-time changes in the carrier’s motion state, related data, and the like.

To meet the diverse requirements for accuracy and robustness in the integrated navigation of UAVs, in light of the navigation algorithm discussed above, this paper incorporates the concept of the interacting multiple model (IMM). According to the different characteristics of each navigation system, appropriate estimation algorithms are adopted, and then an estimation fusion algorithm capable of dynamically matching different conditions is constructed. This is aimed at enhancing the adaptability of the integrated navigation algorithm and providing strong support for the precise navigation of UAVs.

## 2. System Model of SINS/GNSS Integrated Navigation

### 2.1. State Equation for SINS/GNSS Integrated Navigation

The SINS error equation is adopted as the integrated navigation state equation [18], and the navigation frame is defined as the north–east–up (ENU) frame. The mathematical platform error angles $\phi_{E},\phi_{N},\phi_{U}$; velocity errors $\delta v_{E},\delta v_{N},\delta v_{U}$; position errors, including the latitude error, the longitude error, and the altitude error δL,δλ,δh; the constant bias errors of gyroscope $\varepsilon_{x},\varepsilon_{y},\varepsilon_{z}$; and the constant bias errors of accelerometer $\nabla_{x},\nabla_{y},\nabla_{z}$ are selected as the states of the integrated navigation filter:

$${\left[\phi_{E},\phi_{N},\phi_{U},\delta v_{E},\delta v_{N},\delta v_{U},\delta L,\delta \lambda ,\delta h,\;\varepsilon_{x},\varepsilon_{y},\varepsilon_{z},\nabla_{x},\nabla_{y},\nabla_{z}\right]}^{T}$$

Through error source analysis, the integrated navigation system’s state equation can be obtained [19]:

(1) $$\dot{X}(t)=\Phi (t)X(t)+\Gamma (t)W(t)$$

where X(t) denotes state vector; Φ(t) denotes state transition matrix; Γ(t) denotes system noise coefficient matrix; and W(t) denotes system noise vector.

### 2.2. Measurement Equation for SINS/GNSS Integrated Navigation

Taking the position and velocity differences between SINS and GNSS as the measurements, the measurement equation for integrated navigation filter may be characterized as follows [19]:

(2) $$\begin{array}{l} Z(t)=H(t)X(t)+V(t)=\\ \;\left[\begin{array}{c} Z_{p}(t)\\ Z_{v}(t) \end{array}\right]=\left[\begin{array}{c} H_{p}(t)\\ H_{v}(t) \end{array}\right]X(t)+\left[\begin{array}{c} V_{p}(t)\\ V_{v}(t) \end{array}\right] \end{array}$$

where Z(t) denotes the measurement. $Z_{p}(t)$ and $V_{p}(t)$ denote the measurement and noise of the position. $Z_{v}(t)$ and $V_{v}(t)$ denote the measurement and noise of the velocity. $H_{p}(t)$ and $H_{v}(t)$ denote the measurement matrices of position and velocity, respectively.

By performing discretization processing on Formulas (1) and (2), the linear discrete system model shown in Formula (3) can be obtained [19]. Then, an information fusion algorithm is adopted for filter estimation to realize the airborne SINS/GNSS integrated navigation.

(3) $$\left\{\begin{array}{l} X_{k}=\Phi_{k,k-1}X_{k-1}+\Gamma_{k-1}W_{k-1}\\ Z_{k}=H_{k}X_{k}+V_{k} \end{array}\right.$$

## 3. Information Fusion Algorithms and Analysis

### 3.1. Strong Tracking Filter (STF)

The Kalman filter algorithm is well-established and extensively utilized. Hence, its filter recursive equations are directly cited [19,20].

(4) $$\left\{\begin{array}{c} {\hat{X}}_{k/k-1}=\Phi_{k/k-1}{\hat{X}}_{k-1}\\ P_{k/k-1}=\Phi_{k/k-1}P_{k-1}\Phi_{k/k-1}^{T}+\Gamma_{k-1}Q_{k-1}\Gamma_{k-1}^{T}\\ K_{k}=P_{k/k-1}H_{k}^{T}{\left[H_{k}P_{k/k-1}H_{k}^{T}+R_{k}\right]}^{-1}\\ {\hat{X}}_{k}={\hat{X}}_{k/k-1}+K_{k}[Z_{k}-H_{k}{\hat{X}}_{k/k-1}]\\ P_{k}=[I-K_{k}H_{k}]P_{k/k-1} \end{array}\right.$$

When the model of the integrated navigation system and the characteristics of the noise are both accurately known, the optimal state estimation of the integrated navigation can be obtained through the Kalman filter described in Equation (4), which provides a clear mathematical expression and implementation path for the theoretically optimal solution of the integrated navigation.

However, in practical applications, the above-mentioned ideal conditions are usually difficult to meet. Once there is uncertainty within the system model of integrated navigation, or the noise characteristics cannot be accurately known, the performance degradation of the Kalman filter will be significant. The reason for this is that the Kalman filter algorithm exhibits a high dependence on the system model and noise statistical characteristics. Model deviations or non-ideal noise characteristics will interfere with the filter process, thereby resulting in reduced estimation accuracy and degraded stability [21].

To enhance the Kalman filter’s robustness, the strong tracking filter algorithm dynamically adjusts the one-step prediction covariance matrix by introducing the adaptive factor $\lambda_{k}$ [22]:

(5) $$P_{k/k-1}=\lambda_{\;k}\Phi_{k/k-1}P_{k-1}\Phi_{k/k-1}^{T}+\Gamma_{k-1}Q_{k-1}\Gamma_{k-1}^{T}$$

where $\lambda_{k}=\max\left\{\begin{array}{cc} 1, & \frac{tr\left[N_{k}\right]}{tr\left[M_{k}\right]} \end{array}\right\}$, $tr\left[M_{k}\right]$ denotes the trace of $M_{k}$, and $tr\left[N_{k}\right]$ denotes the trace of $N_{k}$.

(6) $$N_{k}={\tilde{C}}_{k}-R_{k}-H_{k}\Gamma_{k-1}Q_{k-1}\Gamma_{k-1}^{T}H_{k}^{T}$$

(7) $${Μ}_{k}=H_{k}\Phi_{k/k-1}P_{k-1}\Phi_{k/k-1}^{T}H_{k}^{T}$$

The adaptive factor can intelligently sense system changes according to the system’s real-time state and measurement, adjust the one-step prediction covariance matrix, and enhance the adaptability and tracking performance of the filter in a complex and changeable environment, thus ensuring state estimation accuracy [23].

The strong tracking filter holds that filter performance degradation results from the mismatch of the state model or the change in process parameters. Therefore, an adaptive factor is introduced to adapt the one-step prediction covariance matrix to mitigate the influence of past and prior data on the current estimation of the state. At the same time, it strengthens the correction of the state estimation using current measurement information, thus enabling the algorithm to quickly track the changes in the measurement information.

However, the strong tracking filter algorithm also has certain limitations: when the measurement information itself is inaccurate, the algorithm will still operate according to its fast tracking mechanism, which will instead lead to a further increase in state estimation error, which impairs the system’s accuracy and reliability.

### 3.2. Smooth Variable Structure Filter Algorithm (SVSF)

In the smooth variable structure filter algorithm, the smooth bounded layer width is introduced. The bounded layer width dictates the average estimation accuracy level and essentially reflects the filter’s interference degree [24].

The calculation formula for smooth bounded layer width can be stated as follows [24]:

(8) $$\psi_{k}={\left[{\left[\mathrm{diag}\left(\left|e_{z,k/k-1}\right|+\gamma \left|e_{z,k-1/k-1}\right|\right)\right]}^{-1}H_{k}P_{k/k-1}H_{k}^{T}S_{k}^{\;-1}\right]}^{-1}$$

where $e_{z,k-1/k-1}=Z_{k-1}-H_{k-1}{\hat{X}}_{k-1}$ denotes the posterior measurement error at the k−1 moment; $e_{z,k/k-1}=Z_{k}-H_{k}{\hat{X}}_{k/k-1}$ denotes the prior measurement error at the k moment; $\gamma \left(0<\gamma <1\right)$ denotes the convergence factor, which affects the convergence rate of the filter; and $S_{k}=E\left[e_{z,k/k-1}e_{z,k/k-1}^{T}\right]=E\left[\left(Z_{k}-H_{k}{\hat{X}}_{k/k-1}\right){\left(Z_{k}-H_{k}{\hat{X}}_{k/k-1}\right)}^{T}\right]=H_{k}P_{k/k-1}H_{k}^{T}+R_{k}$ denotes the measurement error covariance matrix.

With the smooth bounded layer as a basis, the SVSF gain can be calculated [24].

(9) $$K_{k}^{SVSF}=H_{k}^{+}\mathrm{diag}\left[(\left|e_{z,k/k-1}|+\gamma |e_{z,k-1/k-1}\right|)\circ \mathrm{sat}\left(\frac{e_{z,k/k-1}}{\psi_{k}}\right)\right]{\left[\mathrm{diag}\left(e_{z,k/k-1}\right)\right]}^{-1}$$

where “∘” represents the Hadamard product and $\mathrm{sat}\left(\frac{e_{z,k/k-1}}{\psi_{k}}\right)$ indicates to perform a saturation operation on each element in the prior measurement error column vector $e_{z,k/k-1}$:

(10) $$\mathrm{sat}\left(\frac{e_{z,k/k-1}}{\psi_{k}}\right)=\left\{\begin{array}{c} 1,\frac{e_{zi,k/k-1}}{\psi_{kii}}\geq 1\\ \frac{e_{zi,k/k-1}}{\psi_{kii}},-1<\frac{e_{zi,k/k-1}}{\psi_{kii}}<1\\ -1,\frac{e_{zi,k/k-1}}{\psi_{kii}}\leq -1 \end{array}\right.\left(i=1,2,\ldots ,m\right)$$

where $e_{zi,k/k-1}$ denotes the *i*-th element in the prior measurement error column vector $e_{z,k/k-1}$ and $\psi_{kii}$ denotes the *i*-th diagonal element in the smooth bounded width matrix $\psi_{k}$. Through the above $\mathrm{sat}\left(\;\right)$ operation, robustness and stability are ensured outside the smooth bounded layer; within the bounded layer, the filter gain is interpolated to obtain a smooth function to reduce chattering.

When the navigation system is faced with complex working conditions involving interference, the smooth variable structure filter algorithm exhibits good robustness. Through its unique parameter adjustment mechanism, it can effectively resist various types of interference and ensure the stability and reliability of the filter process. However, this algorithm is essentially a suboptimal filter. While enhancing the robustness, it sacrifices the estimation accuracy. This trade-off between accuracy and robustness is an intrinsic attribute in the smooth variable structure filter algorithm. It also poses new research directions for subsequent algorithm improvement and application expansion.

## 4. Proposed Innovative Interactive Robust Filter Framework Fusing STF and SVSF (IF-STF-SVSF)

Based on the previous analysis, the STF is proficient in tracking measurement information and adjusting the one-step prediction covariance matrix in real time. In complex environments with uncertainties in system models and noise, it can still maintain strong robustness and high accuracy. However, for abnormal measurement information, due to the fact that its fast tracking mechanism fails to distinguish the abnormal information, it will still continue to track, ultimately leading to a sharp increase in the error of state estimation. The smooth bounded layer width $\psi_{k}$ of SVSF is a function that includes the measurement variance, measurement matrix, prior measurement error, and posterior measurement error at the previous moment. It is directly related to the quality of the measurement information. This enables the algorithm to exhibit good robustness when faced with abnormal measurement noise, and effectively suppress the interference of abnormal measurements on the filter results.

Based on the complementarity of the applicable conditions of the above two algorithms and by integrating the theory of the interacting multiple model, this paper proposes an interactive robust filter framework fusing STF and SVSF.

The proposed approach assesses input interaction and output interaction based on the STF and the SVSF. STF and SVSF perform parallel filtering on the foundation of the system model established in the “System Model of SINS/GNSS Integrated Navigation” section. The output quantities provided by each sub-filter include the state estimate ${\hat{X}}_{k}$ and its covariance matrix ${\hat{P}}_{k}$, as well as the innovation $r_{k}$ and its covariance matrix $C_{k}$. The likelihood function is calculated according to the innovation $r_{k}$ and its covariance matrix $C_{k}$, and the filter probability $\mu_{k}$ is updated. The filter probability determines the weighting for individual filter in both input interaction and output fusion.

In summary, the detailed workflow of the interactive robust filter algorithm is outlined below:

(1) Input Interaction

Perform interaction, based on the state estimation ${\hat{X}}_{k-1}^{i}$ and covariance ${\hat{P}}_{k-1}^{i}$ of each filter at the previous moment is to obtain the mixed estimation and covariance.

$\mu_{k-1}^{i}$ is the filter probability obtained by the filter i in the moment k−1. $p_{ij}$ is the probability of transitioning from filter i to filter j. The inter-filter transition probability matrix is given by

(11) $$P=\left[\begin{array}{cccc} p_{11} & p_{12} & \cdots & p_{1N}\\ p_{21} & p_{22} & \cdots & p_{2N}\\ \cdots & \cdots & \cdots & \cdots \\ p_{N1} & p_{N2} & \cdots & p_{NN} \end{array}\right]$$

where *N* denotes the total count of sub-filters.

Next, the mixed transition probability from the filter i to the filter j is as follows:

(12) $$\mu_{k-1}^{ij}=\frac{\mu_{k-1}^{i}p_{ij}}{\overset{N}{\underset{i=1}{\Sigma }}\mu_{k-1}^{i}p_{ij}},\left(i,j=1,2,\ldots ,N\right)$$

Then, perform interaction on the estimation results of each sub-filter according to the mixing probability $\mu_{k-1}^{ij}$ to obtain the initial state values of each sub-filter. The initial conditions for the mixed state estimation ${\hat{X}}_{k-1}^{0\;j}$ and the covariance matrix ${\hat{P}}_{k-1}^{0\;j}$ of filter j can be expressed as follows:

(13) $$\left\{\begin{array}{l} {\hat{X}}_{k-1}^{0\;j}=\overset{N}{\underset{i=1}{\Sigma }}{\hat{X}}_{k-1}^{i}\mu_{k-1}^{ij}\\ {\hat{P}}_{k-1}^{0\;j}=\overset{N}{\underset{i=1}{\Sigma }}\mu_{k-1}^{ij}\left\{{\hat{P}}_{k-1}^{i}+\left[{\hat{X}}_{k-1}^{i}-{\hat{X}}_{k-1}^{0\;j}\right]{\left[{\hat{X}}_{k-1}^{i}-{\hat{X}}_{k-1}^{0\;j}\right]}^{T}\right\} \end{array}\right.,\left(j=1,2,\cdots ,N\right)$$

where ${\hat{X}}_{k-1}^{i}$ and ${\hat{P}}_{k-1}^{i}$ represent the state estimate and its covariance matrix for the filter i at the moment of k−1.

(2) Parallel Filter

In this step, the STF and SVSF are implemented in a parallel manner. The initial values of the mixed state estimation ${\hat{X}}_{k-1}^{0\;j}$ and the covariance matrix ${\hat{P}}_{k-1}^{0\;j}$ for the sub-filter j are acquired through input interaction. Coupled with the current measurement information $Z_{k}$ as the input, the model filter is executed in parallel. After executing a complete filter recursion process, the respective state estimation ${\hat{X}}_{k}^{j}$ and its covariance matrix ${\hat{P}}_{k}^{j}$ are obtained.

The SVSF exhibits good robust characteristics against bounded models and noise uncertainties. In addition, the adaptive factor $\lambda_{k}$ in STF can be used to adaptively adjust the system model of the SVSF. Given that the strategy behind the STF model is to dynamically adjust the one-step prediction covariance matrix, the SVSF model adopts the opposite strategy, that is, adjusting the measurement noise covariance matrix to $\lambda_{k}R_{k}$, thereby further adjusting its smooth bounded layer. Thus, the two sub-filters perform parallel filtering based on complementary system models.

According to the filter results, the innovation $r_{k}^{j}$ and its covariance matrix $C_{k}^{j}$ for the sub-filter j may be derived further as follows:

(14) $$\left\{\begin{array}{l} r_{k}^{j}=Z_{k}-H_{k}^{j}{\hat{X}}_{k/k-1}^{j}\\ C_{k}^{j}=H_{k}^{j}P_{k/k-1}^{j}{\left(H_{k}^{j}\right)}^{T}+R_{k}^{j} \end{array}\right.,\left(j=1,2,\cdots ,N\right)$$

(3) Filter Probability Update

The filter probability of the sub-filter j can be updated by calculating its corresponding likelihood function $\Lambda_{k}^{j}$. The likelihood function takes the form of a Gaussian distribution function with respect to the innovation $r_{k}^{j}$ and its covariance matrix $C_{k}^{j}$:

(15) $$\begin{array}{l} \Lambda_{k}^{j}=\frac{1}{\sqrt{{\left(2\pi \right)}^{n}\left|C_{k}^{j}\right|}}\exp\left\{-\frac{1}{2}{\left(r_{k}^{j}\right)}^{T}{\left(C_{k}^{j}\right)}^{-1}r_{k}^{j}\right\},\\ \left(j=1,2,\cdots ,N\right) \end{array}$$

where n denotes the dimensionality of the innovation vector $r_{k}^{j}$.

Then, use the likelihood function $\Lambda_{k}^{j}$ to propagate the filter j’s probability:

(16) $$\mu_{k}^{j}=\frac{\Lambda_{k}^{j}\overset{N}{\underset{i=1}{\Sigma }}\mu_{k-1}^{i}p_{ij}}{\overset{N}{\underset{j=1}{\Sigma }}\Lambda_{k}^{j}\overset{N}{\underset{i=1}{\Sigma }}\mu_{k-1}^{i}p_{ij}},\left(j=1,2,\cdots ,N\right)$$

(4) Output Fusion

According to the updated model probabilities, perform the weighted sum on the state estimations ${\hat{X}}_{k}^{j}$ and the covariance matrices ${\hat{P}}_{k}^{j}$ output by each model sub-filter executed in parallel, and compute for the final state estimation ${\hat{X}}_{k}$ and associated covariance matrix ${\hat{P}}_{k}$ at the current time step:

(17) $$\left\{\begin{array}{l} {\hat{X}}_{k}=\overset{N}{\underset{j=1}{\Sigma }}{\hat{X}}_{k}^{j}\mu_{k}^{j}\\ {\hat{P}}_{k}=\overset{N}{\underset{j=1}{\Sigma }}\mu_{k}^{j}\left\{{\hat{P}}_{k}^{j}+\left[{\hat{X}}_{k}^{j}-{\hat{X}}_{k}\right]{\left[{\hat{X}}_{k}^{j}-{\hat{X}}_{k}\right]}^{T}\right\} \end{array}\right.$$

**Figure 1:**
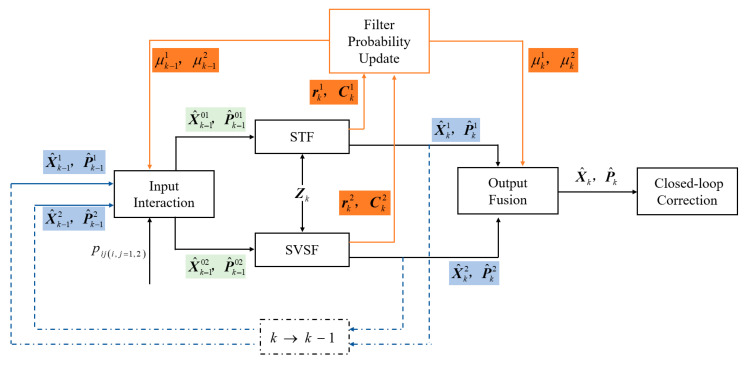
Algorithm structure diagram.

The above four steps loop recursively. The algorithm structure diagram is shown in Figure 1.

The state estimation and covariance matrix obtained by output fusion are used to perform closed-loop correction on the navigation results. In fact, based on the shared strapdown calculation and the integrated navigation model, the interactive filter algorithm runs the STF and SVSF sub-filters in parallel. It updates the filter probabilities on-line in accordance with the sub-filters’ performance metrics, and provides the optimal estimation of the deviation constraint through the likelihood function. In this way, it can obtain navigation results with higher accuracy at a relatively small time cost.

## 5. Experiment and Discussion

To validate the effectiveness of the proposed interactive robust filter algorithm, a simulation platform for the UAV integrated navigation is built to conduct simulation verification, and the results are analyzed.

### 5.1. Experiment Conditions

The initial spatial position takes the value [118.790°, 31.939°, 15 m]. The convergence factor in SVSF is set to γ=0.1. The flight path involves acceleration, pitching, rolling, yawing, and more, covering the conventional maneuvering modes of the UAV. The duration of the flight process is 960 s.

Given that navigation-grade SINS has been successfully applied to large fixed-wing UAVs worldwide, the parameter settings in this study are based on the sensor accuracy of airborne navigation-grade INS. Table 1 specifies navigation sensors parameters for simulation.

To validate the applicability of the proposed algorithm against uncertain system noise and measurement anomalies, artificial inertial measurement unit, and GNSS errors are introduced according to Table 2.

As shown in Table 2, during 100–200 s, GNSS error noise increases. During 500–600 s, inertial measurement unit constant bias increases. During 700–800 s, both GNSS error noise and inertial measurement unit constant bias increase.

### 5.2. Experiment Analysis

The proposed interactive robust filter algorithm, which integrates STF and SVSF, is termed IF-STF-SVSF. Similarly to the idea in Reference [16], the STF and SVSF combined algorithm based on fixed threshold switching is called STF-SVSF. Under the above experiment conditions, comparative analysis is carried out among IF-STF-SVSF, STF, SVSF and STF-SVSF.

**Figure 2:**
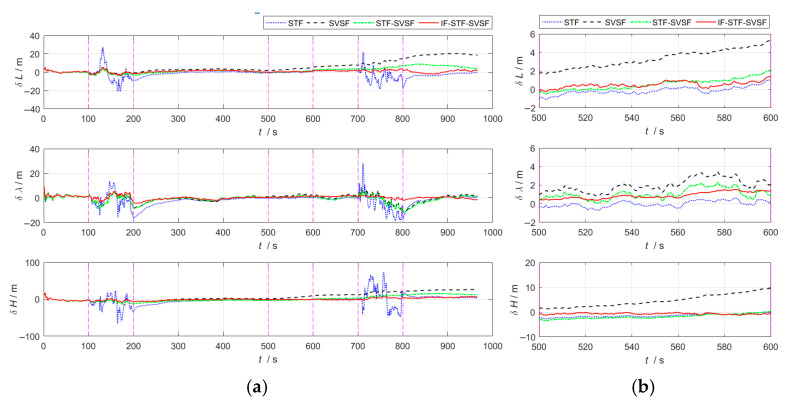
Position error curves for the four algorithms. (**a**) Overall error curves. (**b**) Enlarged curves of 500–600 s.

**Figure 3:**
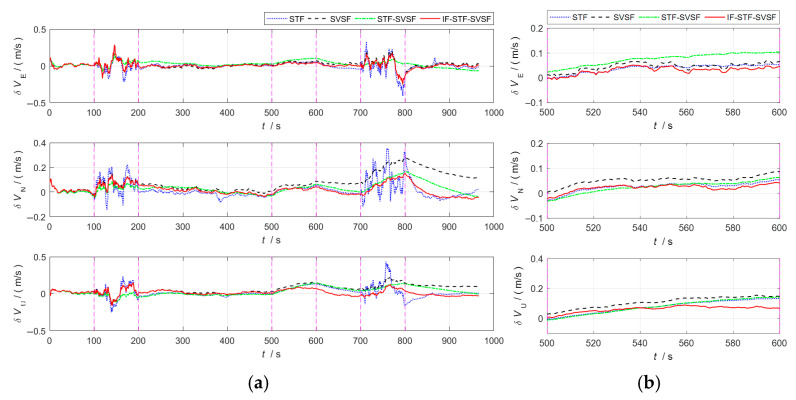
Velocity error curves for the four algorithms. (**a**) Overall error curves. (**b**) Enlarged curves of 500–600 s.

In the integrated navigation process, closed-loop correction is performed on the SINS. Figure 2 and Figure 3, respectively, present the evaluation metrics (position error, velocity error) for the above algorithms during the integrated navigation process.

Figure 2 and Figure 3 demonstrate that, during 100–200 s, the GNSS error noise increases to five times its original level.

(1) The STF tends to trust the measurement information. By introducing a time-varying adaptive factor, it can modulate the one-step prediction covariance matrix in real time, thereby mitigating the influence of prior information on state estimation. Even when faced with abnormal measurement information, this algorithm will quickly track the measurement information, resulting in a substantial rise in state estimation error. Therefore, for integrated navigation system based on STF, both its position error and velocity error show a violently fluctuating trend.
(2) The SVSF adopts the idea of variable structure gain and has good robustness against bounded model and noise uncertainties. Compared with the STF, it shows better adaptability under abnormal measurement information conditions, and the velocity and position accuracy are significantly improved.
(3) The STF-SVSF can switch between the STF and the SVSF according to the system state changes, thereby effectively improving the accuracy of state estimation. However, algorithm switching is limited by the fixed bounded layer threshold, which affects the flexibility and adaptability of the algorithm.
(4) In the proposed IF-STF-SVSF, both the STF and the SVSF dynamically adjust the filter model based on the innovation, calculate and update the filter probability, and solve the weighted state estimation. During the period of GNSS abnormal measurement noise, the SVSF can suppress the innovation mismatch, and its filter probability has a great advantage, effectively improving the estimation accuracy. Therefore, overall, the interactive robust estimation algorithm has the highest fusion accuracy of the four compared methods.

At 500–600 s, the inertial measurement unit constant bias increases to three times its original level.

(1) The STF relies more on measurement information. When the system noise is abnormal, leading to a rise on state estimation error, this algorithm expands one-step prediction covariance matrix according to the innovation mismatch degree, thereby increasing the filter gain. It reduces the usage efficiency of prior information to achieve the purpose of reusing immediate measurement information. Therefore, in the case of changes in the noise characteristics of inertial sensors, the STF can achieve relatively good estimation accuracy.
(2) The SVSF still has good adaptability and robustness when system noise changes and system noise covariance matrix deviate from the true value, and when navigation error is relatively stable. However, since the SVSF sacrifices part of the estimation accuracy for enhanced robustness, its accuracy is inferior to that of the STF.
(3) The STF-SVSF flexibly selects the STF and the SVSF according to the bounded layer threshold, and has a corresponding improvement in state estimation accuracy.
(4) The IF-STF-SVSF can perform real-time calculations based on innovation, update its filter probability, and solve the weighted state estimation. During the period when the inertial measurement unit bias increases, the filter probability of the STF has a major advantage, and the interactive robust estimation algorithm still achieves highest estimation accuracy.

At 700–800 s, the GNSS error noise increases to five times its original level and the inertial measurement unit constant bias increases to three times its original level. Uncertain system noise coincides with anomalies in measurement information.

(1) During the phase where both system and measurement noise increase simultaneously, the performance of the STF is disturbed by the abnormal measurements, and the navigation error shows fluctuations.
(2) The SVSF is not sensitive to the types of anomalies. It has good robust characteristics and strong applicability under bounded model conditions and noise uncertainties. Moreover, it maintains good stability of the navigation error.
(3) The STF-SVSF can flexibly switch between the STF and the SVSF in response to the dynamic changes in the system state, which improves the accuracy of the state estimation.
(4) Integrated navigation based on the IF-STF-SVSF provides the optimal estimation information with bias constraints through the likelihood function during the process of interactive fusion. This makes the estimation residual after fusion decrease further, and its navigation accuracy has more advantages over the comparison algorithms.

**Figure 4:**
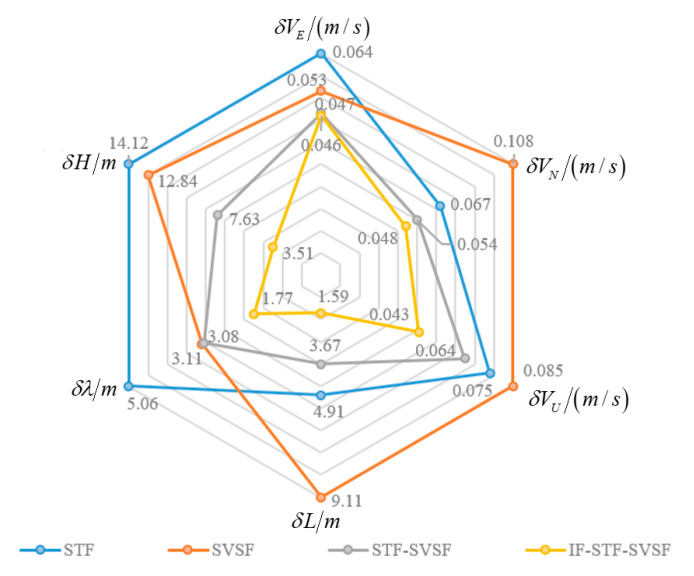
Position and velocity RMSEs the four algorithms.

Furthermore, the Root Mean Square Errors (RMSEs) are adopted as quantitative indicators to verify navigation capability among various comparative methods. Figure 4 presents radar charts of position and velocity RMSEs for the four algorithms mentioned above.

As can be seen from Figure 4, the STF and the SVSF exhibit their respective advantages and disadvantages under different flight states and environments. The STF-SVSF switches between the two algorithms using a fixed threshold, which effectively improves navigation precision. However, the proposed IF-STF-SVSF can dynamically adjust the weights of the two filter algorithms in response to innovation and provide the optimal estimation of bias constraints through the likelihood function during the fusion process, significantly improving the estimation accuracy and robust performance.

The various algorithms are assessed via Matlab R2016a (64-bit, Windows 64), running on the same computer. The filter cycle is set to 1 s. The time consumed per filter period for STF, SVSF, STF-SVSF, and IF-STF-MAKF stands at 10.71 ms, 11.29 ms, 12.24 ms, and 14.81 ms in sequence. It can be observed that the computation times of all four algorithms fall within the same order of magnitude and remain significantly shorter than the filter period. This demonstrates that the proposed algorithm can achieve higher-precision navigation with a relatively small additional time cost.

## 6. Conclusions

To enhance the state estimation performance and robustness of the airborne integrated navigation, this paper investigates an interactive filter algorithm that combines the STF and SVSF. In scenarios where system noise and measurement information are abnormal, this scheme’s feasibility is verified, and its integrated navigation performance is analyzed. The results of simulation experiment demonstrate that the interactive filter proposed herein productively reduces the estimation error. When facing a complex and changeable noise environment and system uncertainties, it can maintain high state estimation accuracy, and its robustness is significantly enhanced. Compared with the STF, SVSF, and STF-SVSF, the velocity accuracy of the proposed interactive robust filter algorithm is improved by more than 16%, and the position accuracy is improved by more than 40%. This method has broad versatility in the integrated navigation system, and it has important reference value for engineering applications. It can also be extended to fields such as land vehicles and mobile robots.

## Figures and Tables

**Table 1:** The parameters of SINS and GNSS.

Sensors	Measurement	Noise	Value
SINS	Gyro	Constant bias	0.01 deg/h
		Angular random walk	0.001 deg/sqrt(h)
	Accelerometer	Constant bias	100 μg
		Velocity random walk	10 μg/sqrt(Hz)
GNSS	Velocity	Error noise	0.1 m/s
	Position	Error noise	10 m, 10 m, 30 m

**Table 2:** The actual errors of the inertial measurement unit and GNSS.

Time (s)	Sensors	Fault Type	Fault Value
100–200	GNSS	velocity error noise	0.5 m/s
		position error noise	50 m, 50 m, 150 m
500–600	SINS	Accelerometer constant bias	300 μg
		Gyro constant bias	0.03 deg/h
700–800	GNSS	velocity error noise	0.5 m/s
		position error noise	50 m, 50 m, 150 m
	SINS	Accelerometer constant bias	300 μg
		Gyro constant bias	0.03 deg/h

## Data Availability

The original contributions presented in this study are included in the article. Further inquiries can be directed to the corresponding author.
